# Prevalence of Low Back Pain and Disc Degeneration in Elite Cross‐Country Skiers: A Comparative Study With Non‐Athlete Controls

**DOI:** 10.1111/sms.70187

**Published:** 2025-12-19

**Authors:** Anni Aavikko, Janne Pesonen, Leena Ristolainen, Niko Murto, Hannu Kautiainen, Teija Lund

**Affiliations:** ^1^ Department of Orthopedics and Traumatology Wellbeing Services County of Päijät‐Häme Lahti Finland; ^2^ Kuopio University Hospital Department of Rehabilitation Kuopio Finland; ^3^ Orton, Research Institute Orton Helsinki Finland; ^4^ Department of Radiology Helsinki University and Helsinki University Hospital Helsinki Finland; ^5^ Primary Health Care Unit, Kuopio University Hospital, Kuopio, Finland and Folkhälsan Research Center Helsinki Finland; ^6^ Department of Orthopaedics and Traumatology Helsinki University and Helsinki University Hospital Helsinki Finland

**Keywords:** athletes, cross‐country skiers, disc degeneration, low back pain, magnetic resonance imaging, non‐athlete controls

## Abstract

Intensive training in competitive sports during childhood and adolescence may increase the risk of low back pain (LBP). While disc degeneration (DD) is a known contributor to LBP, its occurrence among elite cross‐country skiers has not been previously assessed using magnetic resonance imaging (MRI). This study aimed to compare the prevalence of LBP and DD in elite cross‐country skiers and age‐matched non‐athlete controls. Forty‐four elite skiers and 71 controls underwent a structured interview assessing LBP without specific trauma, a clinical examination, and lumbar spine MRI. Skiers also completed patient‐reported outcome measures (PROMs). Disc morphology was evaluated from MRI using the Pfirrmann classification (grades 1–5). A Pfirrmann Summary Score (PSS) was calculated by summing the grades of the individual discs. Analyses were adjusted for age and sex. LBP was reported by 75% of skiers and 54% of controls (*p* = 0.028). DD prevalence, defined as at least one disc graded ≥ 3, did not differ significantly between the groups (50% versus 42%, *p* = 0.78). In both groups, individuals with LBP had significantly higher PSS than their asymptomatic peers. Elite skiers reported more LBP than controls, despite similar DD prevalence. These findings suggest that while intensive training may not increase DD risk, it may contribute to a higher burden of LBP. The association between PSS and LBP was consistent across groups, indicating that structural disc changes may play a role regardless of athletic status.

**Trial Registration:** ClinicalTrials.gov identifier: NCT05641857

## 1 Introduction

Participation in sports during childhood and adolescence is widely recognized as beneficial for its numerous physical and psychological benefits. However, intensive training and early specialization in competitive sports may increase the risk of developing low back pain (LBP) at a young age [1]. A systematic review and meta‐analysis reported a point prevalence of LBP of 42% and a lifetime prevalence of 63% among athletes across 38 different sports [2]. Due to methodological heterogeneity, the authors were unable to identify specific sports with the highest prevalence of LBP, a limitation echoed in other studies comparing different sports disciplines [3]. Two systematic reviews have identified common risk factors for LBP in athletes, including a prior history of LBP and high training volume or intensity [3, 4].

Among elite athletes, cross‐country skiers represent a group with a high prevalence of LBP, although the underlying causes remain poorly understood [5, 6, 7]. Reported prevalence rates of LBP in this population range from 55% to 65% [5, 7, 8]. The prevalence of LBP in the general adolescent population varies widely—from 7% to 72% depending on study design, LBP definition, recall period, and participant age [9].

Structural spinal changes such as intervertebral disc degeneration (DD) and disc herniation are common in the general population and have been associated with LBP in recent meta‐analyses [10, 11]. In several sports, DD appears more prevalent among athletes than non‐athletes [12, 13, 14, 15], although comparisons across sports disciplines are complicated due to differences in physical demands and mechanical loading patterns.

To date, no studies have investigated DD using high‐field magnetic resonance imaging (MRI) in elite cross‐country skiers, nor have they compared the prevalence of LBP and DD between cross‐country skiers and age‐matched controls in the context of modern skiing techniques.

Therefore, the aim of the present cross‐sectional study was to compare the prevalence of LBP and lumbar DD in Finnish elite cross‐country skiers and their age‐matched non‐athlete controls. This study is registered at ClinicalTrials.gov.

## 2 Materials and Methods

### 2.1 Study Design and Participant Recruitment

This cross‐sectional study included elite cross‐country skiers and age‐matched non‐athlete controls. The skier group was recruited from all athletes aged 18 years or older who were selected for the Finnish national cross‐country skiing teams (i.e., national team, challenger group, and junior national team) and who attended team training camps during autumn 2022 (*n* = 23). Additionally, 47 cross‐country skiing athletes aged 17 to 19 years, enrolled in a sports academy with a structured training program and competing at the national level, were invited to participate. In total, 44 of the 70 invited athletes consented to participate.

Information about the study protocol was provided both verbally and in writing during routine team camp meetings by Anni Aavikko and Janne Pesonen. Participation was voluntary, and written informed consent was obtained from all participants. For athletes under 18 years of age, parental consent was also required. All participants were pseudonymized, and data were analyzed using subject‐specific identification numbers. National team coaches were not aware of which athletes participated in the study; moreover, team selections for the season had been finalized prior to study enrollment, ensuring that participation did not influence the selection process.

The study protocol included a structured interview, a clinical examination, patient‐reported outcome measures (PROMs), and lumbar spine MRI.

### 2.2 Control Group

The control group comprised 71 healthy volunteers drawn from a previous longitudinal study. In 1994, healthy 8‐year‐old schoolchildren (*n* = 94) were recruited from six primary schools in the urban capital area of Helsinki to investigate the normal growth and development of the lumbar spine from childhood to early adulthood. The original cohort included 94 participants who were examined at ages 8, 11 (*n* = 81), and 18 (*n* = 71) [11]. For the present study, data from the 18‐ year follow‐up were included. The control participants underwent the same study protocol as the skier group, comprising a structured interview, a clinical examination, and lumbar spine MRI. However, patient‐ reported outcome measures (PROMs) were not collected for the control group.

### 2.3 Structured Interview

For skiers, the structured interview collected the following information: age and general health status, including regular medication use; years of regular cross‐country ski training; annual training volume (hours/year); training volume during competitive and off‐season periods; duration of the training season (months); history of LBP without specific trauma (categorized as past week, past month, past year, or earlier); use of pain medication; history of healthcare visits due to LBP.

For controls, the same questions were asked, excluding those related to ski training. Instead, participants were asked about their general physical activity levels outside of school physical education classes, including which sports disciplines they engaged in, the number of hours per week, and the duration in years.

### 2.4 Clinical Examination

For the control group, height and weight were measured using standard equipment; for skiers, these values were self‐reported. Body mass index (BMI) was calculated using the standard formula: weight in kilograms divided by height squared in meters. A standard musculoskeletal clinical examination was performed for both groups.

### 2.5 Patient‐Reported Outcome Measures (PROMs)

Skiers completed the following PROMs on the day of the clinical examination: EQ‐5D‐5L for quality of life [16], Oswestry Disability Index (ODI) [17] as a symptom‐specific measure of disability in daily activities, and Numeric Rating Scale (NRS, 0–10) for current and past‐week back and neck pain intensity [18].

### 2.6 MRI Investigation

For skiers, lumbar spine MRIs were performed using either a GE Signa HDx or GE Optima MR360 1.5 T scanner (GE Healthcare) with a dedicated spine coil. The following sequences were acquired: T1‐ and T2‐weighted sagittal, STIR sagittal, T2‐weighted axial images. For controls, MRIs were performed using a Siemens Symphony 1.5 T MRI scanner (Siemens) with a dedicated spine coil. Only T2‐weighted sagittal sequences were available from the previous study.

Intervertebral disc morphology from L1/L2 to L5/S1 was assessed on midline sagittal T2‐weighted images using the 5‐grade Pfirrmann classification [19]. Two independent evaluators, a radiologist (Niko Murto) and a spine surgeon (Anni Aavikko), graded the discs while blinded to the participants' clinical data. In cases of disagreement, a third evaluator (Teija Lund) provided a consensus rating. The inter‐rater reliability (ICC) between the two evaluators for all lumbar discs combined was 0.74 (95% CI: 0.65 to 0.85), indicating moderate to good agreement [20].

Discs graded Pfirrmann grade 3 (P3) or higher were considered degenerated. A Pfirrmann summary score (PSS; range 5–25) was calculated by summing the grades of all five lumbar discs [21]. One evaluator (Teija Lund) assessed MRIs in both study groups.

### 2.7 Data Analysis

Group (cross‐country skiers and non‐athlete controls) characteristics were reported as means with standard deviation (SD) for continuous variables and as numbers with percentages (%) for categorical variables. The groups were compared using the t‐test and Pearson's chi‐square test based on their types of data distribution. Monte Carlo *p*‐values (small number of observations) were used for the categorical variables when appropriate. Pfirrmann grade at each lumbar disc level for skiers and controls was analyzed using generalized estimating equations (GEE) between mean of Pfirrmann grade at each lumbar disc level. Relationships between Pfirrmann summary score and LBP were analyzed using two‐way analysis of variance. Intraclass correlation coefficients (ICCs) from a one‐way random model were used to assess inter‐rater reliability. Models included age and sex as covariates when appropriate. The Stata 18.0 (StataCorp LP; College Station, Texas, USA) statistical package was used for the analysis.

## 3 Results

Baseline characteristics of the study population are presented in Table 1. There were no significant differences between the groups except for the mean age (21 vs. 19 years for skiers and controls, *p* < 0.001) and smoking (0% and 18% for skiers and controls, *p* < 0.001).

**TABLE 1:** Characteristics of the study population.

	Controls	Skiers	*p*
	*N* = 71	*N* = 44	
Sex, women, *n* (%)	36 (51)	22 (50)	0.94
Age, years, mean (SD)	19 (1)	21 (4)	< 0.001
BMI, kg/m^2^, mean (SD)	22.9 (4.0)	22.2 (1.7)	0.28
Smoking, *n* (%)	18 (25)	0 (0)	< 0.001
Starting age of active training, years, mean (SD)	—	8.2 (3.5)	—
Practice hours/year, mean (SD)	—	795 (179)	—
Physical activity hours/week, mean (SD)	3.5 (3.6)	—	—
Participation in regular sports activity, *n* (%)	43 (61)	—	—
Endurance sports (e.g., running, skiing), *n*	18	—	—
Contact sports, *n*	7	—	—
Team sports, *n*	14	—	—
Gym training, *n*	16	—	—
Light exercise (e.g., walking, yoga), *n*	7	—	—

LBP was reported by 75% of skiers (*n* = 33/44) and 54% of non‐athlete controls (*n* = 38/71), with the difference reaching statistical significance after adjustment for age and sex (*p* = 0.028). Among the control group, 63% (43/71) engaged in varying levels of physical activity (Table 1).

There was no significant difference in the prevalence of lumbar DD, defined as the presence of at least one disc graded ≥ 3 on the Pfirrmann scale, between skiers (50%) and controls (42%) (*p* = 0.78, adjusted for age and sex). Similarly, the PSS values did not differ significantly between the groups.

**FIGURE 1:**
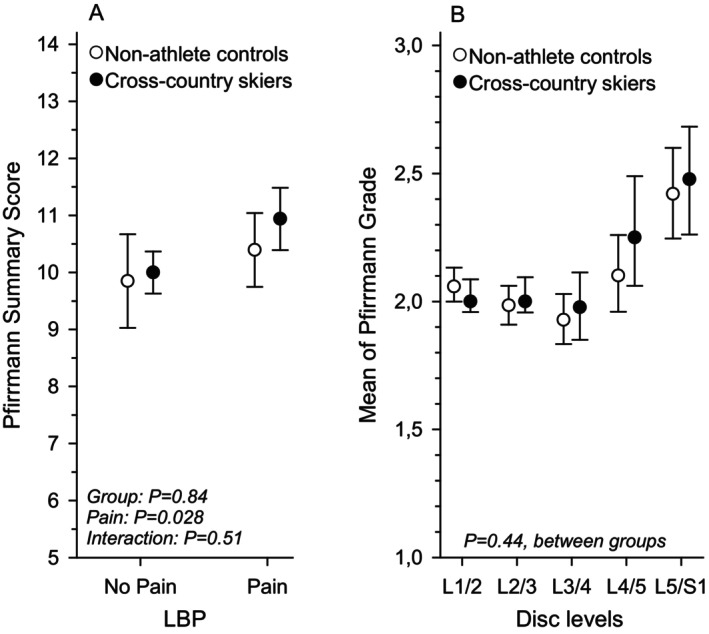
(A) Pfirrmann summary scores in skiers and controls with and without low back pain. (B) Mean of Pfirrmann grade at each lumbar disc level for skiers and controls.

Within both groups, individuals reporting LBP had significantly higher PSS values compared to asymptomatic participants (Figure 1A). Mean Pfirrmann grades at each lumbar disc level for both groups are presented in Figure 1B.

In the clinical examination, the mean finger‐to‐floor distance (FFD) was significantly lower in skiers (mean 1.1 cm, SD 3.2) compared to controls (mean 3.2 cm, SD 6.3) (*p* = 0.006). Hamstring tightness was observed in 18% of skiers and 7% of controls; this difference was not statistically significant (*p* = 0.13).

Among skiers, the mean ODI score was 3.4 (SD 5.1), and the mean EQ‐5D‐5L index value was 0.941 (SD 0.089), reflecting minimal disability and a high level of quality of life.

## 4 Discussion

The primary finding of this study was that elite cross‐country skiers reported a significantly higher prevalence of LBP compared to age‐matched non‐athlete controls, despite no significant difference in the prevalence of lumbar DD between the groups. Although the PSS was higher among individuals with LBP compared to asymptomatic subjects in both groups, no group‐level difference in PSS was observed between skiers and controls. Our findings support the hypothesis that not all LBP in elite cross‐country skiers is discogenic in origin. However, symptomatic individuals in both groups had higher PSS values, suggesting a potential contribution of DD to LBP.

Previous research has suggested that altered biomechanics may contribute to LBP in cross‐country skiers [22, 23]. Modern cross‐country skiing consists of two primary techniques, classic and skating, both commonly used in training and competition. The classic technique comprises three sub‐techniques: double poling (DP), DP with a kick, and diagonal skiing. The skating technique, which is generally more economical and faster, includes four sub‐techniques (V1, V2, V2 alternate, and DP). Skiers switch between these sub‐techniques depending on speed and terrain demands [24].

Advances in skiing technique, equipment, and race formats have increased the physical demands placed on athletes, particularly regarding core strength and trunk mobility [22, 24]. Modern skiing places greater emphasis on force production, acceleration, and speed compared with previous decades [25]. These demands are especially pronounced in the DP and V2 skating techniques [22, 24, 26], which are predominantly used in elite‐level competitions. Over recent decades, DP has evolved into a more dynamic and biomechanically complex movement pattern characterized by increased joint flexion, higher flexion velocities, and greater pole forces during a shorter poling phase [27].

Meeting these technical demands requires increased activation of the erector spinae muscles [28]. Previous studies have associated imbalances in erector spinae activation with LBP in athletes [29], which may partially explain the higher prevalence of LBP observed in contemporary cross‐country skiers. Earlier studies comparing LBP prevalence between cross‐country skiers and controls reported inconsistent findings, likely reflecting differences in technique and race formats before the development of modern skiing [5, 6, 7]. The present study aimed to address this gap by evaluating LBP prevalence in the context of modern cross‐country skiing.

In adolescent cross‐country skiers, rapid increases in training intensity were associated with LBP and secondary scoliosis in boys, both of which improved after correcting technical errors [23]. In elite‐level cross‐country skiers, a correlation has been observed between sagittal balance and LBP, with greater lumbar lordosis relative to thoracic kyphosis linked to a higher likelihood of experiencing LBP [22]. It is important to note that our imaging protocol did not include sagittal balance assessment, limiting our ability to explore this factor further.

Despite early training initiation (mean age 8.2 years), skiers did not exhibit a higher prevalence of DD in early adulthood compared to non‐athlete controls. This contrasts with previous studies reporting increased DD among young athletes [12, 13, 15, 30]. However, methodological differences, such as the use of alternative grading systems (e.g., DeCandido classification [13] and disc signal intensity [12]), may account for some of these discrepancies. Notably, Witwit et al. used the same Pfirrmann classification and found more DD in alpine skiers than controls, despite similar LBP prevalence [15]. The differing mechanical loads in alpine versus cross‐country skiing may explain the contrasting findings between their study and the present study. Axial compression combined with flexion or rotation appears to be particularly detrimental to intervertebral discs (IVDs) [31], whereas high‐volume endurance sports like cycling and running—commonly used as supportive training by skiers—may promote disc health [31, 32].

Our findings were consistent with those of Folkvardsen et al., who reported no difference in the prevalence of DD between elite swimmers and controls at the ages 18 and 20 [33]. Although swimming involves different spinal loading patterns (primary rotation and hyperextension) compared with the flexion and extension patterns typical to cross‐country skiing, the DD prevalence in their study (48% in swimmers versus 53% in controls) was comparable to ours. Similarly, a small cross‐sectional study of female CrossFit athletes found no difference in DD prevalence compared with controls [34].

Thoresson et al. investigated MRI findings and LBP in long‐distance male runners compared with non‐running controls. They reported a significantly higher prevalence of LBP among runners (45% vs. 12%), yet no significant difference in MRI‐detected spinal changes and no correlation between imaging findings and LBP occurrence [35]. These results are consistent with our observations and further suggest that MRI‐identified disc changes do not necessarily explain symptom manifestation in physically active populations.

Importantly, PSS was higher among individuals with LBP in both groups. While previous studies in athletes have not consistently linked DD with LBP [12, 13, 15], such an association has been observed in both adolescent and adult non‐athletes [10, 36]. Our results suggest that the relationship between DD and LBP may be similar in both populations, and that active training does not necessarily influence this association. On the other hand, an equal level of DD may contribute to LBP in athletes compared to non‐athletes due to the greater physical demands placed on their spines.

Contrary to the previous hypothesis, modern cross‐country skiing training does not appear to increase the risk of DD. This is a positive finding, although the high prevalence of LBP among skiers remains concerning. Notably, LBP did not significantly impair their daily functioning, as reflected by low ODI scores.

The relatively small sample size is a limitation of the study. Additionally, 18 athletes completed the clinical examination and interview but did not undergo MRI; among them, 61% (11 of 18) reported LBP, which was lower than the MRI group, though not significantly so. The sample size reflects the highly selective nature of the study population. Elite cross‐country skiers represent a narrow and specialized group, making large‐scale recruitment inherently challenging. Despite the limited number of participants, the sample is representative of an elite‐level athlete population and provides valuable insights into this unique cohort.

Recall bias is always a possibility with self‐reported LBP, though less so for recurrent or severe pain [37]. This is likely less of a concern among elite‐level athletes, who often maintain detailed records of their training and health status. To minimize selection bias, the study was introduced to both the athletes and the non‐athlete controls as a general investigation of lumbar spine health, without specific reference to LBP. Another limitation was the absence of PROM data for the control group, which would have provided valuable insight into the functional impact of LBP across groups. Nonetheless, the control group consisted of healthy volunteers of a similar age to the athletes allowing for a meaningful comparison of structural findings.

Degenerative disc changes were assessed using the Pfirrmann classification. Although this method is based solely on visual grading, it remains widely adopted in research contexts. Incorporating advanced MRI techniques such as T1ρ, T2, and T2* mapping could have enabled the detection of early biochemical alterations that are not apparent on conventional imaging [38]. However, these analyses were not feasible in the present study due to reliance on historical control data.

## 5 Perspective

Early and intensive training does not seem to predispose cross‐country skiers to DD compared with age‐matched non‐athletes. Our findings support the theory that the type of mechanical loading—rather than athletic participation alone—may influence the development of DD in athletes. However, due to the cross‐sectional design of this study, causality between DD, training load, and LBP cannot be inferred. Further research is needed to explore the causes of LBP in athletes and to develop preventive strategies tailored to sport‐specific demands.

## Funding

This work was supported by Research Institute Orton, Helsinki, Finland and Päijät‐Häme Central Hospital, Lahti, Finland through grants by the Ministry of Social Affairs and Health in Finland.

## Ethics Statement

This study was conducted in accordance with the Declaration of Helsinki. Ethical approval was granted by the Ethics Committee of Helsinki University Hospital for skiers on August 3, 2022 (protocol code 3251/2020), and by the Ethics Committee of Orton Orthopedic Hospital for controls on January 22, 1993. The study protocols were approved by the respective Institutional Review Boards. This manuscript adheres to the STROBE guidelines for reporting observational studies where applicable.

## Consent

A written informed consent was signed by all participants and parents from athletes under 18 years of age.

## Conflicts of Interest

The authors declare no conflicts of interest.

## Data Availability

The data that support the findings of this study are available from the corresponding author upon reasonable request.

## References

[1] M. Hangai , K. Kaneoka , Y. Okubo , et al., “Relationship Between Low Back Pain and Competitive Sports Activities During Youth,” American Journal of Sports Medicine 38, no. 4 (2010): 791–796, 10.1177/0363546509350297. 20051500

[2] F. Wilson , C. L. Ardern , J. Hartvigsen , et al., “Prevalence and Risk Factors for Back Pain in Sports: A Systematic Review With Meta‐Analysis,” British Journal of Sports Medicine 55, no. 11 (2021): 601–607, 10.1136/BJSPORTS-2020-102537. 33077481

[3] J. Wall , W. P. Meehan , K. Trompeter , et al., “Incidence, Prevalence and Risk Factors for Low Back Pain in Adolescent Athletes: A Systematic Review and Meta‐Analysis,” British Journal of Sports Medicine 56, no. 22 (2022): 1299–1306, 10.1136/BJSPORTS-2021-104749. 36150752

[4] F. Wilson , C. Gissane , and A. McGregor , “Ergometer Training Volume and Previous Injury Predict Back Pain in Rowing; Strategies for Injury Prevention and Rehabilitation,” British Journal of Sports Medicine 48, no. 21 (2014): 1534–1537, 10.1136/BJSPORTS-2014-093968. 25257230

[5] M. Alricsson and S. Werner , “Young Elite Cross‐Country Skiers and Low Back Pain‐A 5‐Year Study,” Physical Therapy in Sport 7, no. 4 (2006): 181–184, 10.1016/J.PTSP.2006.06.003. 21663829

[6] I. S. Foss , I. Holme , and R. Bahr , “The Prevalence of Low Back Pain Among Former Elite Cross‐Country Skiers, Rowers, Orienteerers, and Nonathletes: A 10‐Year Cohort Study,” American Journal of Sports Medicine 40, no. 11 (2012): 2610–2616, 10.1177/0363546512458413. 22972850

[7] R. Bahr , S. O. Andersen , S. Løken , B. Fossan , T. Hansen , and I. Holme , “Low Back Pain Among Endurance Athletes With and Without Specific Back Loading—A Cross‐Sectional Survey of Cross‐Country Skiers, Rowers, Orienteerers, and Nonathletic Controls,” Spine 29, no. 4 (2004): 449–454, 10.1097/01.BRS.0000096176.92881.37. 15094542

[8] K. Eriksson , G. Németh , and E. Eriksson , “Low Back Pain in Elite Cross‐Country Skiers. A Retrospective Epidemiological Study,” Scandinavian Journal of Medicine & Science in Sports 6, no. 1 (1996): 31–35, 10.1111/J.1600-0838.1996.TB00067.X. 8680941

[9] L. J. Jeffries , S. F. Milanese , and K. A. Grimmer‐Somers , “Epidemiology of Adolescent Spinal Pain: A Systematic Overview of the Research Literature,” Spine 32, no. 23 (2007): 2630–2637, 10.1097/BRS.0B013E318158D70B. 17978666

[10] W. Brinjikji , F. E. Diehn , J. G. Jarvik , et al., “MRI Findings of Disc Degeneration Are More Prevalent in Adults With Low Back Pain Than in Asymptomatic Controls: A Systematic Review and Meta‐Analysis,” AJNR. American Journal of Neuroradiology 36, no. 12 (2015): 2394–2399, 10.3174/AJNR.A4498. 26359154 PMC7964277

[11] A. Aavikko , M. Lohman , L. Ristolainen , et al., “ISSLS Prize in Clinical Science 2022: Accelerated Disc Degeneration After Pubertal Growth Spurt Differentiates Adults with Low Back Pain from their Asymptomatic Peers,” European Spine Journal 31, no. 5 (2022): 1080–1087, 10.1007/s00586-022-07184-0. 35333957

[12] A. Baranto , M. Hellström , C. G. Cederlund , R. Nyman , and L. Swärd , “Back Pain and MRI Changes in the Thoraco‐Lumbar Spine of Top Athletes in Four Different Sports: A 15‐Year Follow‐Up Study,” Knee Surgery, Sports Traumatology, Arthroscopy 17, no. 9 (2009): 1125–1134, 10.1007/S00167-009-0767-3. 19305975

[13] O. Thoreson , P. Kovac , A. Swärd , C. Agnvall , C. Todd , and A. Baranto , “Back Pain and MRI Changes in the Thoraco‐Lumbar Spine of Young Elite Mogul Skiers,” Scandinavian Journal of Medicine & Science in Sports 27, no. 9 (2017): 983–989, 10.1111/SMS.12710. 27367529

[14] K. Lagerstrand , A. Baranto , and H. Hebelka , “Different Disc Characteristics Between Young Elite Skiers With Diverse Training Histories Revealed With a Novel Quantitative Magnetic Resonance Imaging Method,” European Spine Journal 30, no. 7 (2021): 2082–2089, 10.1007/S00586-021-06869-2. 34013394

[15] W. A. Witwit , P. Kovac , A. Sward , et al., “Disc Degeneration on MRI Is More Prevalent in Young Elite Skiers Compared to Controls,” Knee Surgery, Sports Traumatology, Arthroscopy 26, no. 1 (2018): 325–332, 10.1007/S00167-017-4545-3. PMC5754419 28409199

[16] “EQ‐5D User Guides—EQ‐5D,” accessed September 22, 2022, https://euroqol.org/publications/user‐guides/.

[17] L. Pekkanen , H. Kautiainen , J. Ylinen , P. Salo , and A. Häkkinen , “Reliability and Validity Study of the Finnish Version 2.0 of the Oswestry Disability Index,” Spine 36, no. 4 (2011): 332–338, 10.1097/BRS.0B013E3181CDD702. 20823785

[18] J. T. Farrar , J. P. Young , L. LaMoreaux , J. L. Werth , and R. M. Poole , “Clinical Importance of Changes in Chronic Pain Intensity Measured on an 11‐Point Numerical Pain Rating Scale,” Pain 94, no. 2 (2001): 149–158, 10.1016/S0304-3959(01)00349-9. 11690728

[19] C. W. A. Pfirrmann , A. Metzdorf , M. Zanetti , J. Hodler , and N. Boos , “Magnetic Resonance Classification of Lumbar Intervertebral Disc Degeneration,” Spine 26, no. 17 (2001): 1873–1878, 10.1097/00007632-200109010-00011. 11568697

[20] T. K. Koo and M. Y. Li , “A Guideline of Selecting and Reporting Intraclass Correlation Coefficients for Reliability Research,” Journal of Chiropractic Medicine 15, no. 2 (2016): 155–163, 10.1016/j.jcm.2016.02.012. 27330520 PMC4913118

[21] J. H. Määttä , J. I. Karppinen , K. D. K. Luk , K. M. C. Cheung , and D. Samartzis , “Phenotype Profiling of Modic Changes of the Lumbar Spine and Its Association With Other MRI Phenotypes: A Large‐Scale Population‐Based Study,” Spine Journal 15, no. 9 (2015): 1933–1942, 10.1016/J.SPINEE.2015.06.056. 26133258

[22] M. Alricsson , G. Björklund , M. Cronholm , O. Olsson , P. Viklund , and U. Svantesson , “Spinal Alignment, Mobility of the Hip and Thoracic Spine and Prevalence of Low Back Pain in Young Elite Cross‐Country Skiers,” Journal of Exercise Rehabilitation 12, no. 1 (2016): 21–28, 10.12965/JER.150255. 26933656 PMC4771149

[23] K. A. Bergstrøm , K. Brandseth , S. Fretheim , K. Tvilde , and A. Ekeland , “Back Injuries and Pain in Adolescents Attending a Ski High School,” Knee Surgery, Sports Traumatology, Arthroscopy 12, no. 1 (2004): 80–85, 10.1007/S00167-003-0389-0. 14530845

[24] B. Pellegrini , T. L. Stöggl , and H. C. Holmberg , “Developments in the Biomechanics and Equipment of Olympic Cross‐Country Skiers,” Frontiers in Physiology 9 (2018): 9(JUL), 10.3389/FPHYS.2018.00976. 30087621 PMC6066541

[25] T. Stöggl and H. C. Holmberg , “A Systematic Review of the Effects of Strength and Power Training on Performance in Cross‐Country Skiers,” Journal of Sports Science and Medicine 21, no. 4 (2022): 555–579, 10.52082/jssm.2022.555. 36523891 PMC9741725

[26] O. Ohtonen , V. Linnamo , and S. J. Lindinger , “Speed Control of the V2 Skating Technique in Elite Cross‐Country Skiers,” International Journal of Sports Science and Coaching 11, no. 2 (2016): 219–230, 10.1177/1747954116637156.

[27] H. C. Holmberg , S. Lindinger , T. Stöggl , E. Etizolam , and E. Müller , “Biomechanical Analysis of Double Poling in Elite Cross‐Country Skiers,” Medicine and Science in Sports and Exercise 37, no. 5 (2005): 807–818, 10.1249/01.MSS.0000162615.47763.C8. 15870635

[28] M. L. Ohlsson , M. Nilsson , and M. Swarén , “How Does Pole Length Affect Lower Back Muscle Activity at Different Inclines and Skiing Intensities During Double Poling?,” Frontiers in Sports and Active Living 7 (2025): 1438386, 10.3389/FSPOR.2025.1438386. 40040780 PMC11876168

[29] T. Renkawitz , D. Boluki , and J. Grifka , “The Association of Low Back Pain, Neuromuscular Imbalance, and Trunk Extension Strength in Athletes,” Spine Journal 6, no. 6 (2006): 673–683, 10.1016/j.spinee.2006.03.012. 17088198

[30] J. Takatalo , J. Karppinen , S. Näyhä , et al., “Association Between Adolescent Sport Activities and Lumbar Disk Degeneration Among Young Adults,” Scandinavian Journal of Medicine & Science in Sports 27, no. 12 (2017): 1993–2001, 10.1111/SMS.12840. 28075521

[31] D. L. Belavý , M. J. Quittner , N. Ridgers , Y. Ling , D. Connell , and T. Rantalainen , “Running Exercise Strengthens the Intervertebral Disc,” Scientific Reports 7, no. 1 (2017): 1–8, 10.1038/srep45975. 28422125 PMC5396190

[32] D. L. Belavy , M. Quittner , N. D. Ridgers , et al., “Beneficial Intervertebral Disc and Muscle Adaptations in High‐Volume Road Cyclists,” Medicine and Science in Sports and Exercise 51, no. 1 (2019): 211–217, 10.1249/MSS.0000000000001770. 30157104

[33] S. Folkvardsen , E. Magnussen , J. Karppinen , et al., “Does Elite Swimming Accelerate Lumbar Intervertebral Disc Degeneration and Increase Low Back Pain? A Cross‐Sectional Comparison,” European Spine Journal 25, no. 9 (2016): 2849–2855, 10.1007/S00586-016-4642-X. 27289544

[34] M. Wegner , J. C. Backhauß , Y. Michalsky , et al., “Prevalence of Degenerative Vertebral Disc Changes in Elite Female Crossfit Athletes—A Cross‐Sectional Study,” BMC Musculoskeletal Disorders 24, no. 1 (2023): 963, 10.1186/S12891-023-07071-9. 38082262 PMC10712126

[35] O. Thoreson , K. Svensson , P. Jonasson , P. Kovac , L. Sward , and A. Baranto , “Back Pain and MRI Abnormalities in the Thoraco‐Lumbar Spine of Elite Long Distance Runners A Cross Sectional Study,” Medical Research Archives 2, no. 4 (2015): 325.

[36] M. M. van den Heuvel , E. H. G. Oei , S. M. A. Bierma‐Zeinstra , and M. van Middelkoop , “The Prevalence of Abnormalities in the Pediatric Spine on MRI: A Systematic Review and Meta‐Analysis,” Spine 45, no. 18 (2020): E1185–E1196, 10.1097/BRS.0000000000003527. 32355138

[37] S. Milanese and K. Grimmer‐Somers , “What Is Adolescent Low Back Pain? Current Definitions Used to Define the Adolescent With Low Back Pain,” Journal of Pain Research 3 (2010): 57–66, 10.2147/jpr.s10025. 21197310 PMC3004638

[38] R. U. Din , X. Cheng , and H. Yang , “Diagnostic Role of Magnetic Resonance Imaging in Low Back Pain Caused by Vertebral Endplate Degeneration,” Journal of Magnetic Resonance Imaging 55, no. 3 (2022): 755–771, 10.1002/JMRI.27858. 34309129

